# Association of Hemorrhoids With Hashimoto's Thyroiditis and Associated Comorbidities: A Nationwide Population-Based Cohort Study

**DOI:** 10.3389/fendo.2020.577767

**Published:** 2020-10-08

**Authors:** Sheng-Pang Hsu, Hsin-Hung Chen, Tzu-Yuan Wang, Chun-Wei Ho, Ming-Chia Hsieh, Hei-Tung Yip, Chia-Hung Kao

**Affiliations:** ^1^Division of Endocrinology and Metabolism, Department of Internal Medicine, China Medical University Hospital, Taichung, Taiwan; ^2^Intelligent Diabetes Metabolism and Exercise Center, China Medical University Hospital, Taichung, Taiwan; ^3^School of Medicine, Chung Shan Medical University, Taichung, Taiwan; ^4^Division of Endocrinology and Metabolism, Department of Internal Medicine, Asia University Hospital, Taichung, Taiwan; ^5^Department of Internal Medicine, College of Medicine, China Medical University, Taichung, Taiwan; ^6^Division of Endocrinology, China Medical University Hospital, Taichung, Taiwan; ^7^Graduate Institute of Integrative Medicine, China Medical University, Taichung, Taiwan; ^8^Division of Clinical Nutrition, China Medical University Hospital, Taichung, Taiwan; ^9^Management Office for Health Data, China Medical University Hospital, Taichung, Taiwan; ^10^College of Medicine, China Medical University, Taichung, Taiwan; ^11^Graduate Institute of Biomedical Sciences, College of Medicine, China Medical University, Taichung, Taiwan; ^12^Center of Augmented Intelligence in Healthcare, China Medical University Hospital, Taichung, Taiwan; ^13^Department of Nuclear Medicine and PET Center, China Medical University Hospital, Taichung, Taiwan; ^14^Department of Bioinformatics and Medical Engineering, Asia University, Taichung, Taiwan

**Keywords:** hemorrhoids, hashimoto autoimmune thyroiditis, national health insurance research database (NHIRD), cohort study, comorbidity

## Abstract

**Background:** To evaluate the relationship between hemorrhoids and Hashimoto's thyroiditis (HT).

**Methods:** Using Taiwan's Longitudinal Health Insurance Database, we compared the incident risk of HT between the study cohort (comprising patients with hemorrhoids) and the comparison cohort (comprising patients without hemorrhoids). Both cohorts were followed from index date until the date of HT diagnosis, withdrawal from the National Health Insurance program, or the end of 2015.

**Results:** The study cohort and comparison cohort comprised 6,486 patients with hemorrhoids and 25,944 patients without, respectively. The mean follow-up time was ~3 years. The incidence rate of HT in the study cohort was 5.37 per 1,000 person-years, which was higher than that of the control cohort (2.46 per 1,000 person-years). The risk of developing HT in the study cohort was 2.06 times (95% confidence interval [CI] = 1.02, 4.19) higher than that in the comparison cohort.

**Conclusion:** In our study, patients with hemorrhoids could be at increased risk of HT compared with patients with other comorbidities of HT, such as cardiovascular disease.

## Introduction

Hemorrhoids are commonly diagnosed in general health checkups (1) and are an increasingly common gastrointestinal disorder. Many clinical manifestations such as asymptomatic or rectal bleeding result in poor quality of life (2). Risk factors for hemorrhoids include high intra-abdominal pressure and fragile supporting structure. Many conditions such as obesity, constipation, diarrhea, chronic or persistent cough, pregnancy or delivery, and prolonged standing may increase intra-abdominal pressure (3, 4). Studies since the 1980's have noted a shift in the population group with high incidence of hemorrhoids from elderly to middle-aged patients. Various reasons have been offered for this, including changes in nutritional habits (4, 5). Hospital-based proctoscopy studies have reported that the incidence of hemorrhoids could be as high as 86%, but most patients with hemorrhoids are asymptomatic (6).

Hashimoto's thyroiditis (HT) was first described in Japan in 1912 by Dr. Hakaru Hashimoto (7). HT is part of the spectrum of chronic autoimmune thyroid diseases associated with varying degrees of hypothyroidism. Many thyroid autoantibodies for HT have been identified, including thyroid peroxidase antibodies and thyroglobulin antibodies. The prevalence of HT is dependent on age, gender, and race. For example, HT is most prevalent in the 45–55 age group. Women are 4–10 times more likely to have HT than men, and HT is more common in whites than in blacks, Hispanics, or Asians. Systemic manifestations of HT originate from loss of thyroid function. The signs and symptoms of hypothyroidism vary across most organs and tissues. Constipation is the most common complaint reported in patients with hypothyroidism (8–10). To our best knowledge, limited research exists on the correlation between hemorrhoids and HT, although hemorrhoids and HT have the same signs and symptoms, such as constipation. We analyzed a nationwide population-based dataset to determine the association between hemorrhoids and HT.

## Methods

### Data Source

The National Health Insurance Research Database (NHIRD) contains medical information on almost all Taiwanese residents. Data have been collected since 1995, upon the launch of the compulsory single-payer National Health Insurance (NHI) program. The Longitudinal Health Insurance Database (LHID) contains data from one million randomly selected beneficiaries. We used the outpatient, inpatient, and medication records in the LHID to study the relationship between hemorrhoids and HT. Diagnoses and prescriptions were recorded according to the diagnostic codes of the *International Classification of Disease, Ninth. Revision, Clinical Modification* (*ICD-9-CM*). This study was approved by the Research Ethics Committee of China Medical University Hospital (CMUH104-REC2-115-CR-4).

### Study Population

Patients with two or more outpatient diagnoses of or one hospitalization for hemorrhoids (*ICD-9-CM* codes 455.0–455.5) in the period of 2000–2004 were recruited as the study cohort, and patients without hemorrhoids comprised the comparison cohort. We excluded patients who were diagnosed as having HT prior to entry into the study, patients aged under 18 years, or patients with one episode of hemorrhoids. Four patients in the comparison cohort were matched to one patient in the study cohort according to sex, age, and year of entry. The participants were followed up until December 31, 2015. Patients who died or withdrew from the NHI program during the study were considered to be censored.

### Outcome Measurement and Covariates

The primary end-point of this study was the diagnosis of HT (*ICD-9-CM* code 245.21), defined as having at least two outpatient visits or one admission record. The associated comorbidities were coronary artery disease (*ICD-9-CM* codes 410–414), heart failure (*ICD-9-CM* code 428), diabetes (*ICD-9-CM* code 250), depression (*ICD-9-CM* codes 269.2, 296.3 300.4, 311), stroke (*ICD-9-CM* codes 430–438), hypertension (*ICD-9-CM* codes 401–405), hyperlipidemia (*ICD-9-CM* code 272), chronic kidney disease (*ICD-9-CM* codes 580–589), and constipation (*ICD-9-CM* code 564.0). Treatment of hemorrhoids was also considered to be a confounder.

### Statistical Analysis

The statistical significance of differences between categorical and continuous variables between the study cohort and the comparison cohort was evaluated using the chi-square test and Student's *t*-test, respectively. The univariable Cox proportional hazards model was used to estimate the hazard ratio and the 95% confidence interval (CI). The multivariable Cox proportional hazards model was then used to obtain the adjusted hazard ratio. We used the Kaplan–Meier method to quantify the cumulative incidence curve and examined it using the log-rank test. All analyses were generated using SAS software, Version 9.4 (SAS Institute Inc., Cary, NC, USA). A *p* < 0.05 was considered statistically significant.

## Results

The study cohort comprised 6,486 patients with hemorrhoids, and the comparison cohort comprised 25,944 patients without hemorrhoids. The mean follow-up time was ~3 years. The differences in gender and age between the cohorts were accounted for by matching. Table 1 shows the distribution of the baseline characteristics of the cohorts. With respect to gender and age, most participants were men (57%) and were aged over 50 years (39%). Patients with comorbidities presented more frequently in the study cohort than in the comparison cohort.

**Table 1:** Baseline characteristics of patients with and without hemorrhoids.

	**Hemorrhoids**				
	**No (*****N*** **=** **25,944)**		**Yes (*****N*** **=** **6,486)**		
**Variables**	***n***	**%**	***n***	**%**	***p*-value**
Gender					1.00
Female	11,196	43%	2,799	43%	
Male	14,748	57%	3,687	57%	
Age, year					1.00
18–29	3,820	15%	955	15%	
30–39	5,776	22%	1,444	22%	
40–49	6,168	24%	1,542	24%	
≥50	10,180	39%	2,545	39%	
Mean, (SD)	47.5	(16.5)	47.6	(16.5)	0.77
**Comorbidities**					
CAD	453	2%	171	3%	<0.001
heart failure	583	2%	202	3%	<0.001
DM	2,379	9%	724	11%	<0.001
Depression	821	3%	385	6%	<0.001
Stroke	1,638	6%	504	8%	<0.001
Hypertension	5,197	20%	1,599	25%	<0.001
Hyperlipidemia	2,849	11%	995	15%	<0.001
CKD	881	3%	309	5%	<0.001
Constipation	1,901	7%	1,227	19%	<0.001

^*^Evaluated by t-test.

*CAD, coronary artery disease; DM, diabetes mellitus; CKD, chronic kidney disease*.

*Follow-up: comparison cohort: 3.45 (1.52); study cohort: 3.44 (1.52)*.

**Figure 1:**
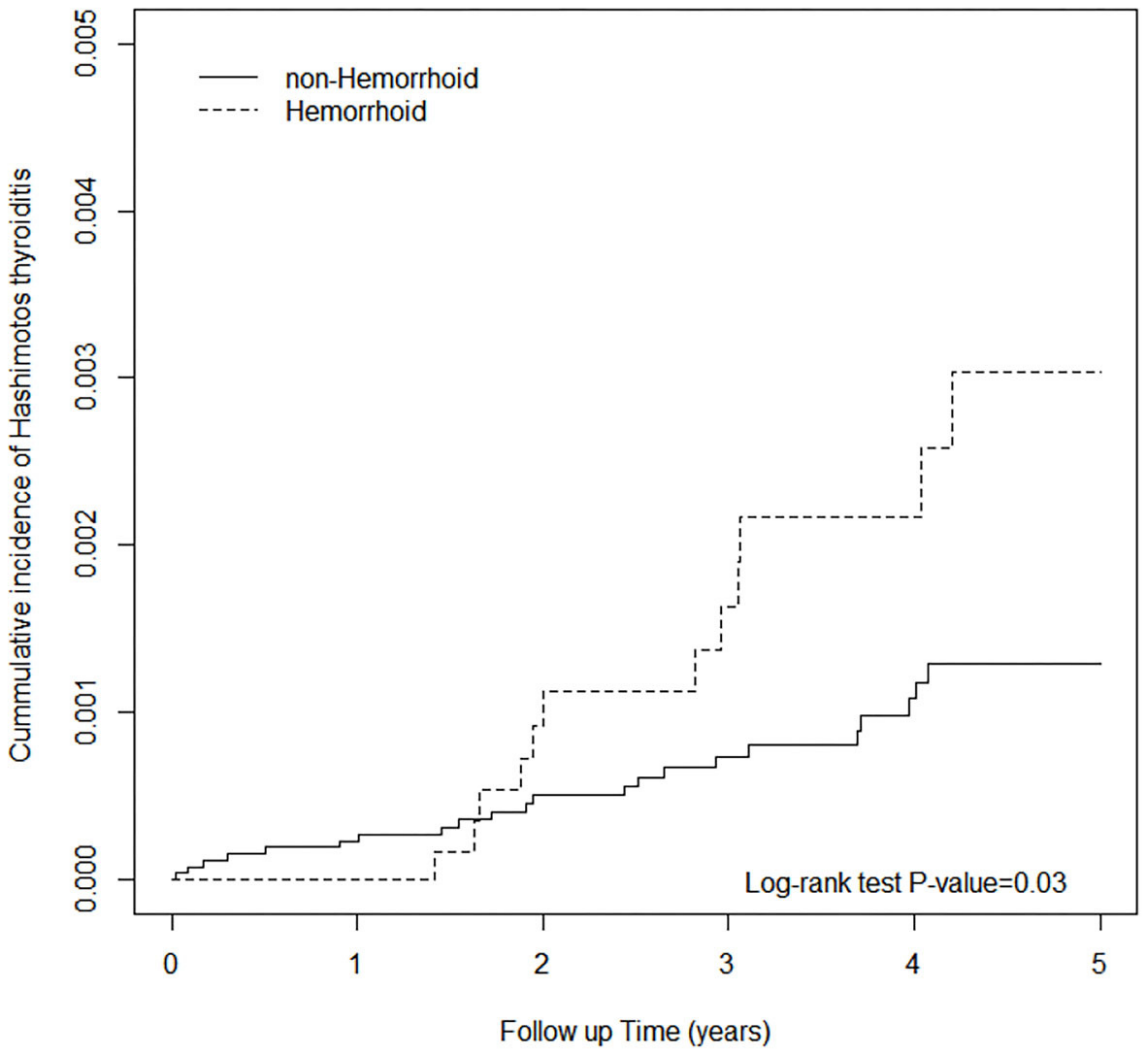
Cumulative incidence of Hashimoto's thyroiditis between individuals with and without hemorrhoids.

The results of the Cox proportional hazards analysis are presented in Table 2. The incidence rate of HT in the study cohort was 5.37 per 1,000 person-years, which was considerably higher than that of the comparison cohort (2.46 per 1,000 person-years). Compared with patients without hemorrhoids, after controlling for gender and age, patients with hemorrhoids were 2.06 times more likely (95% CI = 1.02, 4.19) to develop HT. Figure 1 shows that the cumulative incidence of HT in the study cohort was higher than that in the comparison cohort after the 2nd year of follow-up. Female patients were more likely to have HT than male patients (adjusted hazard ratio = 0.17, 95% CI = 0.07, 0.40). Patients with CAD increase the risk of HT by 4.72 times (95% CI = 1.04, 21.4) relative to those without CAD. In Table 3, the effect of hemorrhoid treatment on Hashimotos thyroiditis was not observed.

**Table 2:** Incidence rates and hazard ratios of Hashimoto's thyroiditis.

	**Hashimotos thyroiditis**						
**Variables**	***n***	**PY**	**IR**	**cHR**	**(95% CI)**	**aHR^†^**	**(95% CI)**
Non-hemorrhoids	22	89,447	2.46	1.00	(Reference)	1.00	(Reference)
Hemorrhoids	12	22,344	5.37	2.18	(1.08, 4.41)^*^	2.06	(1.02, 4.19)^*^
**Gender**							
Female	28	48,344	5.79	1.00	(Reference)	1.00	(Reference)
Male	6	63,447	0.95	0.16	(0.07, 0.39)^***^	0.17	(0.07, 0.40)^***^
**Age, year**							
18–29	8	16,709	4.79	1.00	(Reference)	1.00	(Reference)
30–39	7	25,293	2.77	0.58	(0.21, 1.59)	0.64	(0.23, 1.77)
40–49	9	26,363	3.41	0.71	(0.27, 1.84)	0.77	(0.29, 1.99)
≥50	10	43,426	2.30	0.48	(0.19, 1.21)	0.49	(0.19, 1.30)
**Comorbidities**							
**CAD**							
No	32	109,779	2.91	1.00	(Reference)	1.00	(Reference)
Yes	2	2,012	9.94	3.45	(0.83, 14.4)	4.72	(1.04, 21.4)^*^
**Heart failure**							
No	34	109,331	3.11	1.00	(Reference)		
Yes	0	2,460	0.00	0.00	(0, Inf)		
**DM**							
No	32	101,649	3.15	1.00	(Reference)		
Yes	2	10,142	1.97	0.63	(0.15,2.63)		
**Depression**							
No	31	108,044	2.87	1.00	(Reference)	1.00	(Reference)
Yes	3	3,747	8.01	2.85	(0.87, 9.34)	2.22	(0.66, 7.46)
**Stroke**							
No	31	104,685	2.96	1.00	(Reference)		
Yes	3	7,106	4.22	1.43	(0.44, 4.69)		
**Hypertension**							
No	25	89,453	2.79	1.00	(Reference)		
Yes	9	22,338	4.03	1.44	(0.67, 3.09)		
**Hyperlipidemia**							
No	29	99,763	2.91	1.00	(Reference)		
Yes	5	12,028	4.16	1.46	(0.56, 3.77)		
**CKD**							
No	33	108,076	3.05	1.00	(Reference)		
Yes	1	3,715	2.69	0.90	(0.12, 6.57)		
**Constipation**							
No	30	102,072	2.94	1.00	(Reference)		
Yes	4	9,719	4.12	1.44	(0.51,4.08)		

* *p < 0.05*;

*** *p < 0.001*.

*PY, person-years; IR, incidence rate per 10,000 person-years; cHR, crude hazard ratio; aHR, adjusted hazard ratio; CI, confidence interval; CAD, coronary artery disease; DM, diabetes mellitus; CKD, chronic kidney disease*.

† *adjusted for gender, age, CAD, and depression*.

**Table 3:** Association between hemorrhoid treatment and Hashimoto's thyroiditis.

		**Hashimotos thyroiditis**						
**Hemorrhoids**	**Drug**	***n***	**PY**	**IR**	**cHR**	**(95% CI)**	**aHR^†^**	**(95% CI)**
No	No	22	89,447	2.46	1.00	(Reference)	1.00	(Reference)
Yes	No	8	15,677	5.10	2.08	(0.93, 4.68)	2.09	(0.93, 4.69)
Yes	Yes	4	6,667	6.00	2.42	(0.83, 7.02)	2.41	(0.83, 7.00)

*PY, person-years; IR, incidence rate per 10,000 person-years; cHR, crude hazard ratio; aHR, adjusted hazard ratio; CI, confidence interval*.

† *adjusted for gender and age*.

## Discussion

Our mean analysis, including that of the participants' baseline characteristics, was consistent with analyses in previous research (2, 3), indicating the representativeness of our study cohort. The patients with hemorrhoids in our study all had comorbidities such as constipation, cardiovascular disease, diabetes, and depression. Studies have demonstrated the association between hemorrhoids and cardiovascular disease (11, 12). Multiple risk factors have been identified for hemorrhoids in adults, including age, pregnancy, abdominal obesity, depression, constipation, prolonged sitting on the toilet, lack of dietary fiber consumption, and inadequate hydration (3, 4, 13). Our analysis revealed similar results; for example, constipation and depression were more prevalent in the study cohort than in the comparison cohort. The risk of developing HT was 2.06 times higher in patients with hemorrhoids than in patients without hemorrhoids. The relationship between hemorrhoids and HT stratified by gender was not significant. This contrasts with past studies, including our own, indicating that female patients are more likely to have HT than are male patients (8–10). In addition, one study noted that constipation and positive family history are the primary risk factors for external hemorrhoidal disease in children and teenagers (14). In our study, there was no any significant difference in younger or older patients with hemorrhoid. Constipation was another confounding factor to be analyzed in our study. We did not use the patients aged <18 years due to the complete data for adults and we are doctors for internal medicine instead of pediatrics.

Our study indicates several possible explanations. HT is the most common endocrine disorder and the most common cause of hypothyroidism (8, 15, 16). It leads to multi-systemic manifestations: constipation and reduced peristalsis (gastrointestinal); increased cholesterol (endocrine); obstruction of the upper airways by enlarged soft tissue, causing chronic or persistent coughing (pulmonary); bradycardia, decreased ventricular contractility, and increased peripheral resistance (cardiovascular); liquid retention or dehydration from decreased glomerular filtration (urinary); and inability to concentrate, memory loss, and depression (neuropsychiatric) (8). Our previous study also identified an association between depression and HT (17). Another study reported that HT causes insulin resistance and metabolic syndrome (18) and noted the role of obesity in insulin resistance and hypothyroidism (19). In hemorrhoids, increased intra-abdominal pressure is a primary cause and also results in strenuous bowel movements. Hemorrhoids are prevalent among patients with obesity and may increase stress on the rectal muscle. Physical inactivity might also contribute to the development of hemorrhoids because of blood stasis in the pelvis (20, 21). Obesity was also identified as a risk factor for hemorrhoids in the Korea National Health and Nutrition Examination Survey (22). All these factors may be associated with HT. Indeed, there was no pathogenetic link between hemorrhoids and HT. We used the big data analysis to find the association for these two diseases based on the basic knowledge of thyroid physiology. Again, our health insurance data could not offer the lab data of thyroid function such as TSH, FT4, or T3 and thyroid anti-body such as anti-TPO, and we only used the other clues to approve the association between hemorrhoid and HT including the status of hypothyroidism, subclinical hypothyroid disease, or euthyroidism. Although our sample size of the cohort is relatively large, the number of patients with HT was still very small because of the low incidence of HT. This might cause the less statistical power to any meaningful analyses with stratification. Our finding only provides the possibility of the association between hemorrhoid and HT. Further basic study for the real mechanism of these two diseases is needed in the future.

## Limitations

Our study has some limitations. First, the encoded data for hemorrhoids, HT, and the comorbidities were completely in accordance with ICD codes. Second, patient data on family history, nutritional status, alcohol consumption, cigarette smoking, body mass index, physical activity, and psychological stress were not available in the NHIRD. Third, some laboratory data, including data on thyroid function, thyroid antibodies, and thyroid ultrasound images, were also unavailable due to database restrictions. Fourth, patient data on severity of hemorrhoids were not available in the database.

## Conclusion

In our study, patients with hemorrhoids could be at increased risk of HT compared with patients with other comorbidities of HT, such as cardiovascular diseases. Our findings offer the view to make HT screening for the specific health checkups to patients with hemorrhoid and cardiovascular disease (1). Public health policy could focus on preventing future development of other comorbidities in such patients. Further large-scale studies are required to confirm the clinical significance of our findings.

## Data Availability Statement

The datasets presented in this article are not readily available because the dataset used in this study is held by the Taiwan Ministry of Health and Welfare (MOHW). The Ministry of Health and Welfare must approve our application to access this data. Any researcher interested in accessing this dataset can submit an application form to the Ministry of Health and Welfare requesting access. Please contact the staff of MOHW (Email: stcarolwu@mohw.gov.tw) for further assistance. Taiwan Ministry of Health and Welfare Address: No.488, Sec. 6, Zhongxiao E. Rd., Nangang Dist., Taipei City 115, Taiwan (R.O.C.). Phone: +886-2-8590-6848. All relevant data are within the paper. Requests to access the datasets should be directed to stcarolwu@mohw.gov.tw.

## Ethics Statement

The studies involving human participants were reviewed and approved by This study was approved by the Research Ethics Committee of China Medical University Hospital (CMUH104-REC2-115-CR-4). Written informed consent for participation was not required for this study in accordance with the national legislation and the institutional requirements.

## Author Contributions

All authors: conception and design, administrative support, collection and data assembly, data analysis and interpretation, manuscript writing, and final approval of manuscript.

## Conflict of Interest

The authors declare that the research was conducted in the absence of any commercial or financial relationships that could be construed as a potential conflict of interest.

## Abbreviations

HT: Hashimoto's thyroiditis
CIs: confidence intervals
NHIRD: National Health Insurance Research Database
LHID 2000: Longitudinal Health Insurance Database 2000
ICD-9-CM: International Classification of Diseases, Ninth Revision, Clinical Modification.
